# A Rotational Cylindrical fMRI Phantom for Image Quality Control

**DOI:** 10.1371/journal.pone.0143172

**Published:** 2015-12-01

**Authors:** David A. Tovar, Wang Zhan, Sunder S. Rajan

**Affiliations:** 1 Division of Biomedical Physics, Food and Drug Administration, Silver Spring, Maryland, United States of America; 2 Maryland Neuroimaging Center, University of Maryland, College Park, Maryland, United States of America; North Shore Long Island Jewish Health System, UNITED STATES

## Abstract

**Purpose:**

A novel phantom for image quality testing for functional magnetic resonance imaging (fMRI) scans is described.

**Methods:**

The cylindrical, rotatable, ~4.5L phantom, with eight wedge-shaped compartments, is used to simulate rest and activated states. The compartments contain NiCl_2_ doped agar gel with alternating concentrations of agar (1.4%, 1.6%) to produce T_1_ and T_2_ values approximating brain grey matter. The Jacard index was used to compare the image distortions for echo planar imaging (EPI) and gradient recalled echo (GRE) scans. Contrast to noise ratio (_CNR_) was compared across the imaging volume for GRE and EPI.

**Results:**

The mean T_2_ for the two agar concentrations were found to be 106.5±4.8, 94.5±4.7 ms, and T_1_ of 1500±40 and 1485±30 ms, respectively. The Jacard index for GRE was generally found to be higher than for EPI (0.95 versus 0.8). The CNR varied from 20 to 50 across the slices and echo times used for EPI scans, and from 20 to 40 across the slices for the GRE scans. The phantom provided a reproducible CNR over 25 days.

**Conclusions:**

The phantom provides a quantifiable signal change over a head-size imaging volume with EPI and GRE sequences, which was used for image quality assessment.

## Introduction

Blood oxygenated level dependent (BOLD) functional magnetic resonance imaging (fMRI) is a widely used neuroimaging modality, and researchers and clinicians continue to explore its possible applications. Presently, fMRI is used to study a variety of questions, ranging from visual object recognition [1] to various aspects of cognition [2]. Likewise, clinical fMRI research includes a wide breadth of diseases including schizophrenia [3], Alzheimer’s disease [4], Parkinson’s disease [5], epilepsy [6], and presurgical planning [7] amongst others. However, technical challenges associated with fMRI have restricted the clinical use of fMRI [8–10]. Obtaining robust fMRI signal activation also requires overcoming noise from many different sources such as physiology [11, 12], motion artifacts [13], and instrumentation [12]. Because the results obtained from fMRI studies are based on statistically derived activation maps, the presence of instrumental noise could contribute to inaccurate activation maps.

Instrumental noise is independent of subject variability and can systematically affect large sets of data, leading to possible erroneous results and conclusions. Instrumental noise in the context of fast scanning as with typical echo planar imaging (EPI) arises from both the inherent noise that is present in the absence of image artifacts, as well as the contributions from image artifacts (such as ghosting). One of the main challenges in fMRI is the need to detect relatively small changes in signal. The typical EPI signal change from resting state to active state seen in an fMRI is 1–5% at 3 Tesla [10].

In addition to image noise, image distortion also contributes to degradation of image quality during EPI. The gradient system performance (temporal and linearity), static magnetic field inhomogeneities, and pulse-sequence implementation can contribute to distortion [14]. Gradient coil nonlinearities can cause “most’ distortion along the outer regions of the imaging volumes, while shim inhomogeneity can result in mis-locations of nearly 5 voxels. It has also been reported that gradient intensive scanning can lead to spatial and temporal drift of the magnetic field [15]. Therefore, assessing image quality and the ability to detect small changes in T2*-based signal, in a head-size phantom could be valuable in the implementation and quality control of fMRI.

A number of fMRI phantoms have been previously reported to assess the instrumental noise in fMRI. The Friedman-Glover phantom [16] uses mean signal-to-noise ratio (SNR), mean signal fluctuation noise ratio, mean percent fluctuation, and mean drift, but does not address the ability to detect signal change needed in an fMRI paradigm. Dynamic phantoms such as the SMART phantom [17], and Olsrud phantom [18] bring the added benefit of simulating signal change as in an fMRI paradigm. A simulated signal change in the phantom allows for assessment of contrast-to-noise ratio (CNR), a single and intuitive parameter to estimate the quality of a functional data set [19]. However, dynamic phantoms introduce additional difficulties. The Olsrud phantom contains an asymmetric dynamic imaging volume of 820 ml, and suffers from B_0_ inhomogeneity effects during the fMRI scan. Also, the volume studied is small relative to an average human brain [20]. The SMART phantom requires the use of customized radiofrequency resonators that are switched repeatedly to simulate an fMRI scan. Hence, this design may be difficult to implement in most MRI scanners. Also, a relatively small region within the phantom can be studied using this method. Since ultra-fast scanning methods are used in BOLD fMRI, there is a need for the assessment of both the spatial characteristics (over a large volume) as well as the temporal characteristics.

Building on the work of previous fMRI phantoms, we have constructed a novel cylindrical phantom, with eight segmented compartments (wedges) containing agar gel that allows for evaluation of image quality and distortion over a head-size imaging volume. Rotating the phantom by 45° back and forth simulated “active” and “resting” states (two rotational states). The images from the two rotational states were used to study the temporal and spatial CNR characteristics by pixel-wise computation.

Although, CNR quality was the main objective of this phantom, the segmented design also allowed comparison of the spatial distortions that are seen with the use of the EPI using Jaccard index. Jaccard index which is the ratio of the size of the intersection (of two images: A∩B) to the union (of the same two images: A∪B) provides a coarse measure of distortion of the test image A to undistorted image B. The Jaccard index has been used as an exclusion metric in multi-subject fMRI data [21] as well as to improve spatial registration in fMRI and DTI [22]. In this study the Jaccard index is used to estimate spatial distortion in EPI images relative to magnetization-prepared rapid gradient echo (MPRAGE)

The stability of this phantom design was also tested over several weeks on one MRI scanner. In addition, contributions from coil characteristics, phantom heterogeneity and gradient hardware were probed by comparing images from spin-echo and conventional gradient echo imaging.

## Methods

### Construction of wedge phantom

**Fig 1:**
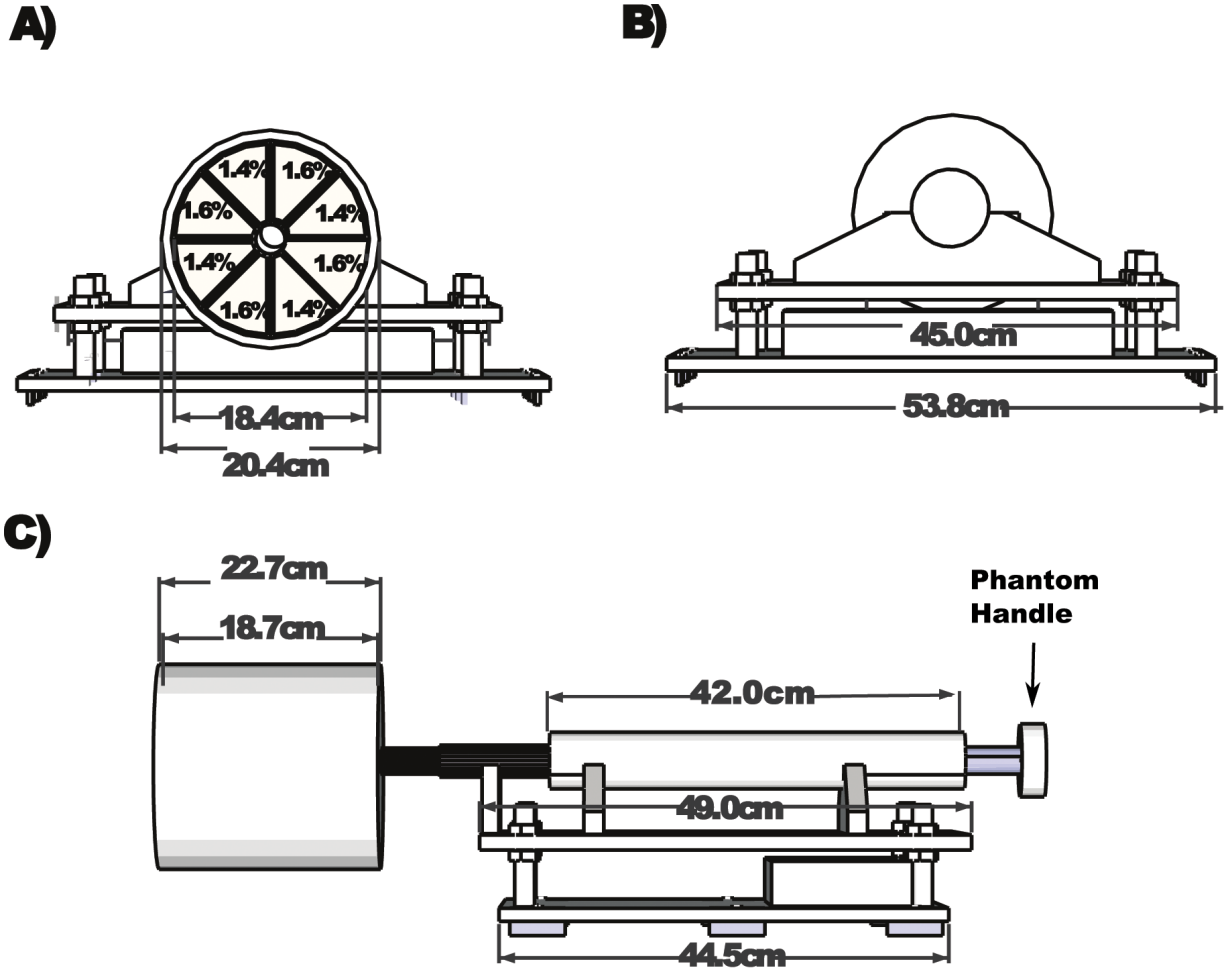
Front and Side view of the cylindrical phantom. Top left: Front view of phantom/ Alternating Agar concentrations are labeled for the wedge compartments. Diameter dimensions of head phantom, which is inserted into a head coil are shown. Top right: Back view of phantom with handle. Handle turns 45 degrees clockwise and counter clockwise creating two “states” during fMRI experiments. Bottom: Side view of phantom. The lower base inserts and thus locks into patient table.

The phantom was composed of a lower base, upper turning base, and a segmented cylinder as shown in Fig 1. The 44.5 cm long lower base included inserts, which fit onto slots along the MR patient table. The 49.0 cm long upper turning base has a 42 cm long hollow cylinder mounted on it. A shaft connecting the head phantom (segmented cylinder) runs through the hollow cylinder and allows the head phantom to be rotated manually. The rotation angle is controlled to be 45 degrees using screw-stops on the shaft.

The cylindrical head phantom dimensions are; acrylic cast thickness: 1 cm, outer- length/diameter: 22.7/20.4 cm, volume 4.6 L. The cylinder was divided with acrylic partitions into eight wedge-shaped compartments, connected to a center column, as seen in Fig 1. The phantom was large enough to measure scanner performance over a field of view exceeding that of the size of a typical human brain. The compartments were filled with alternating concentrations of nickel chloride doped agarose gel (as described in the next section). Thus, during a typical EPI based fMRI scan protocol (eg. 60 volume scans), the phantom was rotated by +45° after the 15^th^ scan, then back by -45° after the 30^th^ scan, and rotated again by +45° after the 45^th^ scan. During such a paradigm, every voxel will generate an alternating signal simulating the “rest” and “active” signal change of an fMRI block design experiment.

### Gel preparation

Agar gels were made by heating a slurry of presoaked (24 hr) agar (Fisher Scientific, Hampton, NH, USA) and nickel chloride (Sigma-Aldrich, St. Louis, MI) in a stainless steel container, with mechanical stirring, until the temperature increased to 100°C in the gel [23]. The gels were made in several batches, half of them contained 0.4 mM NiCl_2_ and 1.4% agar and the other half contained 0.4 mM NiCl_2_ and 1.6% agar. The phantom compartments were then filled in alternating fashion with 1.4% and 1.6% agar gel. The relaxation times of this gel mixture approximated that of brain grey matter at 3T (T_1_ ≈1500ms and T_2_ ≈100ms). In addition, the slightly different agar concentrations used in the adjacent compartments provided a small difference in the T2 signal to mimic the resting and active states in fMRI [24, 25].

### Scan procedures

A 3T Siemens Trio MRI system with a 12-channel head coil was used. Two types of scans were performed: 1) Measurement of the T_1_ and T_2_ distribution of the agarose gel over the volume of the phantom under static conditions. 2) Measurement of signal changes while the phantom was rotated within the MRI bore to mimic an fMRI scan. With the exception of a multi-echo spin echo scan, all scans had an axial slice to slice coverage of 165mm.

A magnetization-prepared rapid gradient echo (MPRAGE) scan (TR/TE/FA = 1900 ms/ 2.26 ms/9°, matrix = 256x224, voxel size = 1 x 1 x 1 mm) provided the baseline images to align the slices of all subsequent scans. An EPI based inversion recovery pulse sequence (TR/TE/FA = 15000 ms/81 ms/90°, matrix = 192x192, 30 slices, voxel size = 1.2 x 1.2 x 5.0 mm) at sixteen inversion times from 50-5000ms was used for T_1_ measurements. At the same slice positions, a fast-spin-echo (FSE) scan (TR/TE/FA = 5000 ms/154 ms/120°, matrix = 448x448, slices = 30 slices, voxel size = 0.5 x 0.5 x 5 mm) was performed to collect T_2_-weighted images. A single-slice, axial, multi-spin-echo (TR/TE = 1000 ms/ 15 ms to 480 ms/, matrix = 128x128, voxel size = 1.76 x 1.76 x 5 mm) at three slice positions along the z-axis of the phantom (0, ±5 cm) was used to measure T_2_ relaxation times to demonstrate the T_2_ differences within the compartments.

Gradient-echo EPI (TR/TE/FA = 4000 ms/21 ms-115 ms/90°, matrix = 64x64, 30 slices, voxel size = 3.52 x 3.52 x 5 mm) was used to test image quality using simulated block design fMRI experiments. Each slice position matched the slice location for the T_1_ and T_2_ scans. A total of 60 dynamic scans were collected for each fMRI run. To obtain simulated functional maps, the phantom was manually rotated clockwise after the 15^th^ scan, counter-clockwise after the 30^th^ scan, and clockwise after the 45^th^ scan. Thus, for each EPI time series, the phantom was rotated three times, to produce a four-block design (two “resting” blocks, two “active” blocks). Each turn rotated the phantom forty-five degrees, and every counter-rotation rotated the phantom back to its initial position. For the beginning of each run, the phantom was returned to the same initial starting position. The simulated fMRI run was repeated using eight different echo times (TE): 21, 30, 40, 50, 60, 80, 100, and 115 ms to measure the CNR for the two rotational states as a function of TE. In addition to EPI, a standard gradient echo scan (GRE) (TR/TE/FA = 1880 ms/ 30 ms/90°, matrix = 128x128, 30 slices, voxel size = 1.76 x 1.76 x 5 mm,) was repeated 4 times, with a rotation after the 2^nd^ repetition.

Note that the slice location and slice spacing were maintained constant throughout a number of scans: inversion recovery, turbo spin echo, echo planar imaging, and gradient echo scans. This was done to compare the T_1_ and T_2_ properties of the phantom to the functional maps obtained by rotating the phantom. This scanning protocol was repeated during three separate sessions over the course of four weeks on the same 3T Siemens Trio MR system.

### Analysis

The image files were exported as DICOM to an off-line workstation and converted to Nifti format (Neuroimaging Informatics Technology Initiative File) for further analysis. Images were used without corrections for distortion or intensity scaling.

MATLAB^®^ (MathWorks Natwick, MA) and the public domain software “AFNI” [26, 27], along with in-house scripts were used to perform image processing. Voxels outside the image were excluded by a masking algorithm created using the AFNI program 3dROIMaker [27]. To minimize partial volume effects near the partitions, a six-parameter rigid body volume registration to correct for roll, pitch, yaw, and translations was performed using the AFNI 3dvolreg program [28]. The first time point image was used as the reference image. The voxels from the partition edges were eliminated, by eroding one voxel away from the edge of the original mask used to isolate the phantom voxels. This operation further reduced possible partial volume effects that might arise from phantom rotation.

#### T1 and T2 maps

Relaxation time maps were computed on a voxel wise basis from the signal intensities and inversion times using a mono-exponential fit, as per Eqs (1) and (2) [29, 30].

$$\text{S}\left(\text{TI}\right)\;=\;|{\text{S}}_{\text{o}}\left(1-2\alpha \;e^{\left(-\;\frac{\text{TI}}{T_{1}}\right)}+e^{\left(-\;\frac{\text{TR}}{T_{1}}\right)}\right)|.$$ (1)

$$\text{S}(\text{TE})=\;{\text{S}}_{\text{o}}e^{\left(-\frac{TE}{\text{T}2}\right)}.$$ (2)

Where S(TI) is the voxel signal for a given inversion time TI and S(TE) is the voxel signal for a given TE.

#### Jaccard analysis

A group-to-reference approach, in which a group of images is registered to a single reference image, was used to measure potential spatial warping of the EPI and GRE images. The center slice of the MPRAGE image set was used as the reference image after resampling to equalize the matrix sizes. The voxels outside the phantom were isolated by using an intensity threshold. The Jaccard index *J*, defined as the intersection of two images, divided by their union was then calculated for each EPI slice with respect to the reference using Eq (3).

$$J\left(A,B_{z}\right)=\;\frac{A\cap B_{z}}{A\cup B_{z}}.$$ (3)

Where, ‘A’ represents the center MPRAGE slice while, ‘B_z_’ represents each of the EPI slices.

#### Contrast to Noise Ratio (CNR)

To assess the simulated signal change caused by phantom rotation, CNR maps were created for each EPI and GRE run (a run defined as a set of dynamic images simulating an fMRI scan, 60 dynamic EPI, 4 acquisition blocks and 4 GRE scans, 2 acquisition blocks). CNR was computed according to Eq (4) where S_A_ and S_B_ refer to the signal from the two rotational states of the phantom and σ is the temporal standard deviation.

$$\text{CNR}=\frac{{\text{S}}_{\text{A}}-{\text{S}}_{\text{B}}}{\sigma }.$$ (4)

For dynamic EPI runs, the mean of the signal for the two rotational states (A: volumes 1–15 and 31–45, B: volumes 16–30 and 46–60) were computed for each voxel, after excluding the transition volumes (1, 15, 16, 30, 31, 45, 46 and 60). The temporal standard deviation (σ) for each voxel was calculated by first computing the standard deviation of the voxels from each image acquisition block (S_A1_, S_B1_, S_A2_, S_B2_) and then computing the mean of these four values. The CNR value for each voxel was computed using Eq (4) from the means ($\bar{S_{A}}$, $\bar{S_{B}}$) and σ.

The CNR for the GRE scans was calculated from the two S_A_ and two S_B_ volumes. An alternate approach was used to estimate σ [31]. The signal intensity of each voxel subtracted by its respective mean ($\bar{S_{A}}$ or $\bar{S_{B}}$) was taken to calculate the residual for each voxel. The four residuals were used to compute σ. As in the EPI scans, the CNR was calculated for each voxel using Eq (4).

To summarize the voxel-wise CNR data and develop potential metrics for assessing fMRI scans, mean and standard deviation of the CNR for each slice was calculated from voxel values (CNR_mean_ and CNR_std_). The standard deviation of the CNR was integrated into a second metric, coefficient of variation (CV_CNR_), given by the ratio of CNR_std_ to CNR_mean_.

## Results

**Fig 2:**
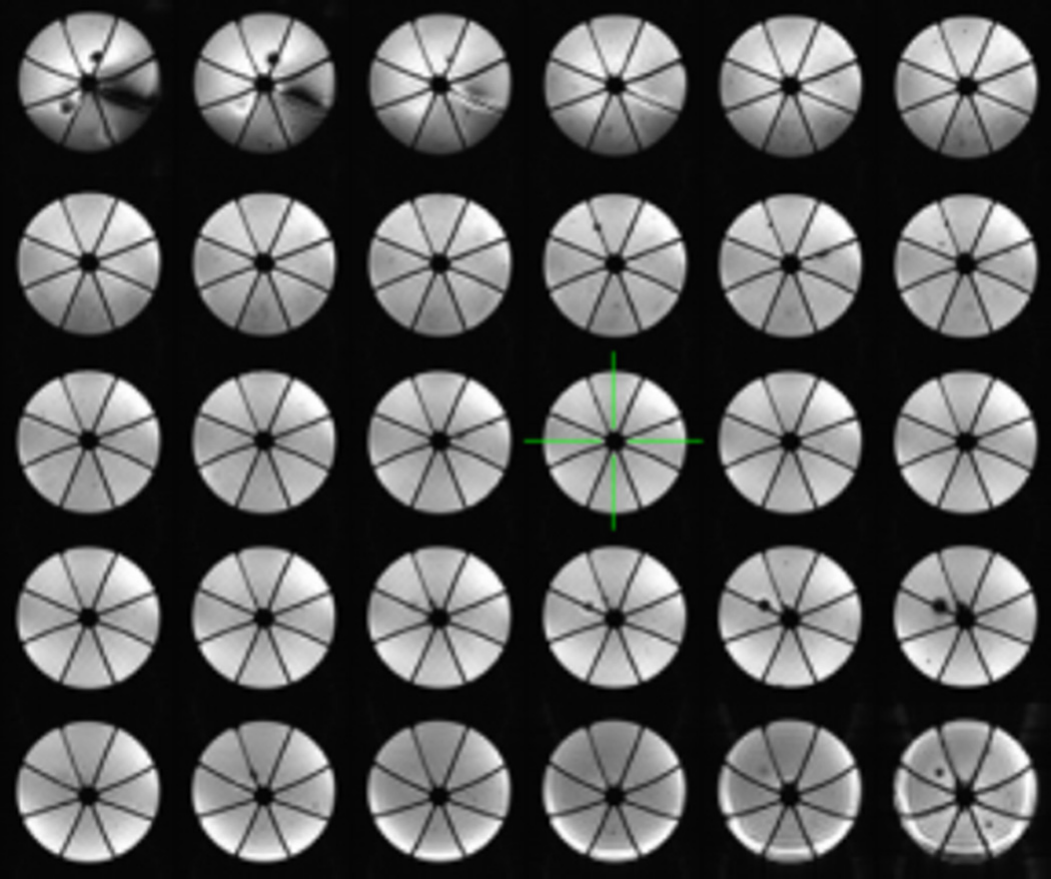
An example of 2D EPI image set obtained using the phantom. TR/TE/FA = 4000ms/30ms/90°, matrix = 64x64, number of slices = 30, voxel size = 3.52mm x 3.52mm, slice thickness = 5 mm. The outer slices show distortion as evidenced by the curved partitions and signal loss due to the drop off in coil sensitivity.

Fig 2 shows an example of an image set (TE = 30 ms) of one of the 60 acquisitions from a typical EPI run. The images show an acceptable coverage volume over the phantom. Each image shows the eight compartments, with the outer slices showing distortion, as evidenced by the curved partitions.

### Relaxation time distribution in the phantom

The mean T_2_ values measured at three slice positions is shown in Table 1, demonstrating clear separation for the two agar concentrations (e.g. In slice #16, the center slice the T_2_ values are: 108.01±5.55 ms versus 94.47± 5.77 ms for 1.4% and 1.6% agar concentrations, respectively). Mean T_1_ values measured using the inversion recovery EPI were found to be 1500 ± 40 ms and 1470 ± 30 ms for regions with 1.4%, 1.6% agar, respectively. The similar value of T_1_ from the two compartments ensures that EPI contrast will be determined by the T_2_ values.

**Table 1:** Mean T_1_/T_2_ values measured at three different slice positions from the two adjacent wedge compartments containing 1.4% and 1.6% agar.

Slice Position(cm)	1.4% agar (ms)	1.6% agar (ms)
**-5 (slice 21)**	1378.04±46.70/104.68±4.09	1357.49±57.60/90.95±3.98
**0 (slice 16)**	1357.49±57.60/108.01±5.55	1331.85± 37.31/94.47± 5.77
**+5 (slice 11)**	1372.23±27.08/106.84±4.68	1372.23± 33.98/98.08± 4.23

**Fig 3:**
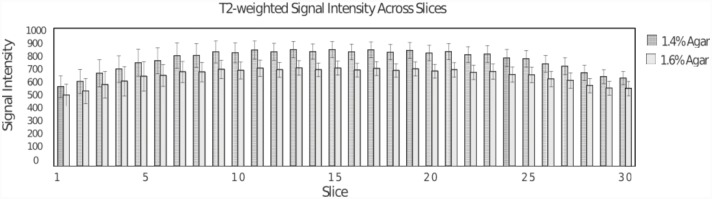
Signal behavior within and across slices from TSE scan (TE = 154 ms). For each slice, the mean value for the 1.4% agar compartment is slightly higher than that for the 1.6% agar concentration as expected. The signal behavior reflects the coil sensitivity as well as the phantom uniformity.

Fig 3 shows the distribution of signal using the FSE sequence across the phantom, for the two agar compartments. There is considerable signal drop-off observed at the extreme slice locations (1–3, 29–30) due to the expected coil sensitivity (~25%). The signal distribution across the slices demonstrates that the phantom material is relatively homogenous. The signal intensity for 1.4% agar (dark grey bars) and for 1.6% (light grey bars) in the center slice was found to be 890±122 and 740±102 arbitrary units.

### Phantom motion effects

The total displacement in roll, pitch, yaw, superior-inferior translation, left-right translation, and posterior-anterior translation was found to be less than 5mm for each scan. The roll correction did not exceed 3mm, demonstrating that the phantom position did not substantially deviate from its initial starting position during manual rotations.

### Jaccard index

**Fig 4:**
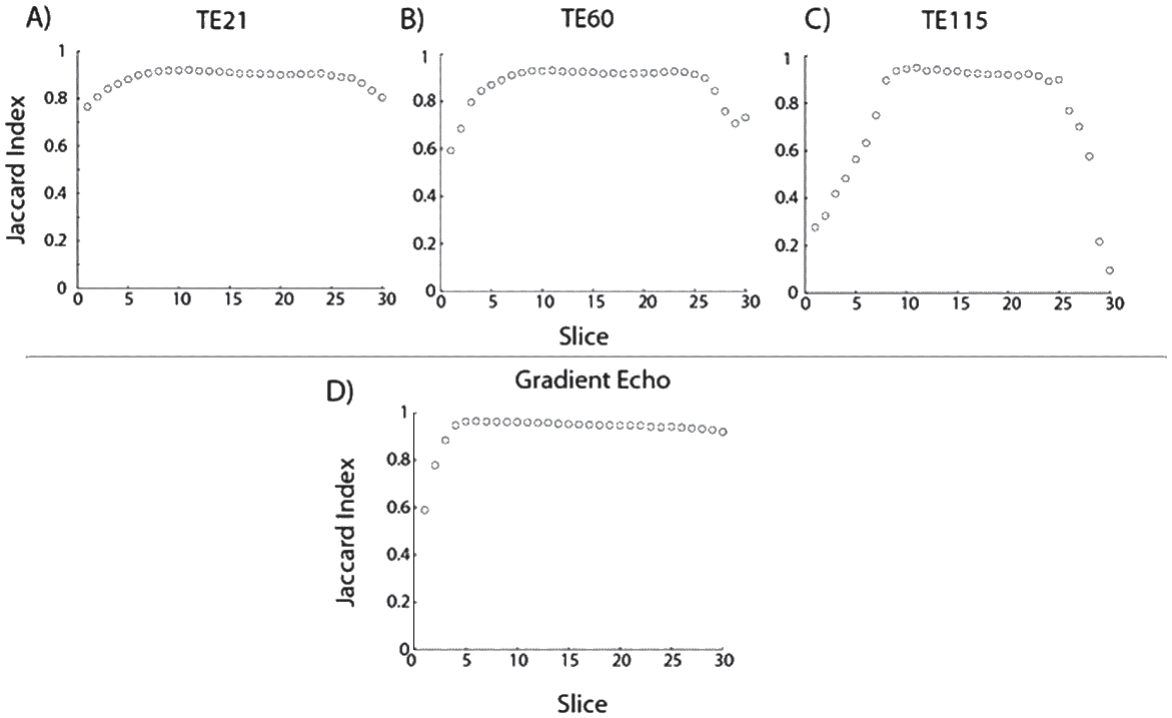
Plot of Jaccard index across slices. The index values represents the relative warping of the various images compared to the rescaled center slice MPRAGE image. A value of 1 represents minimal deviation compared to the corresponding MPRAGE image. A) EPI TE = 21ms, B) EPI TE = 60 ms, C) EPI TE = 115ms, and D) GRE TE = 30ms. A significant drop off in the index is seen in the outer slices of the EPI images, which worsens with longer echo times as expected.

Fig 4 demonstrates the computed Jaccard index, a measure of warping relative to the MPRAGE image, for EPI and GRE scans. The GRE scan as seen in Fig 4D shows the least amount of distortion with Jaccard indexes above 0.95 for all, except the first three slices. Fig 4A, 4B and 4C illustrate that warping effects along the edge slices increases with TE (21, 60 and 115 ms) in the EPI scans as expected. In Fig 4A, all but the first two slices show an index value of > 0.8, with distortion being generally worse than the GRE scan. Upon increasing the TE to 60 ms (Fig 4B), distortion has greatly increased in the first six slices (Jaccard index < 0.4), and now including inferior slices as well. This trend continues at TE of 115ms (Fig 4C), showing further drop in the index across the slices.

### Signal change between the rotational states

**Fig 5:**
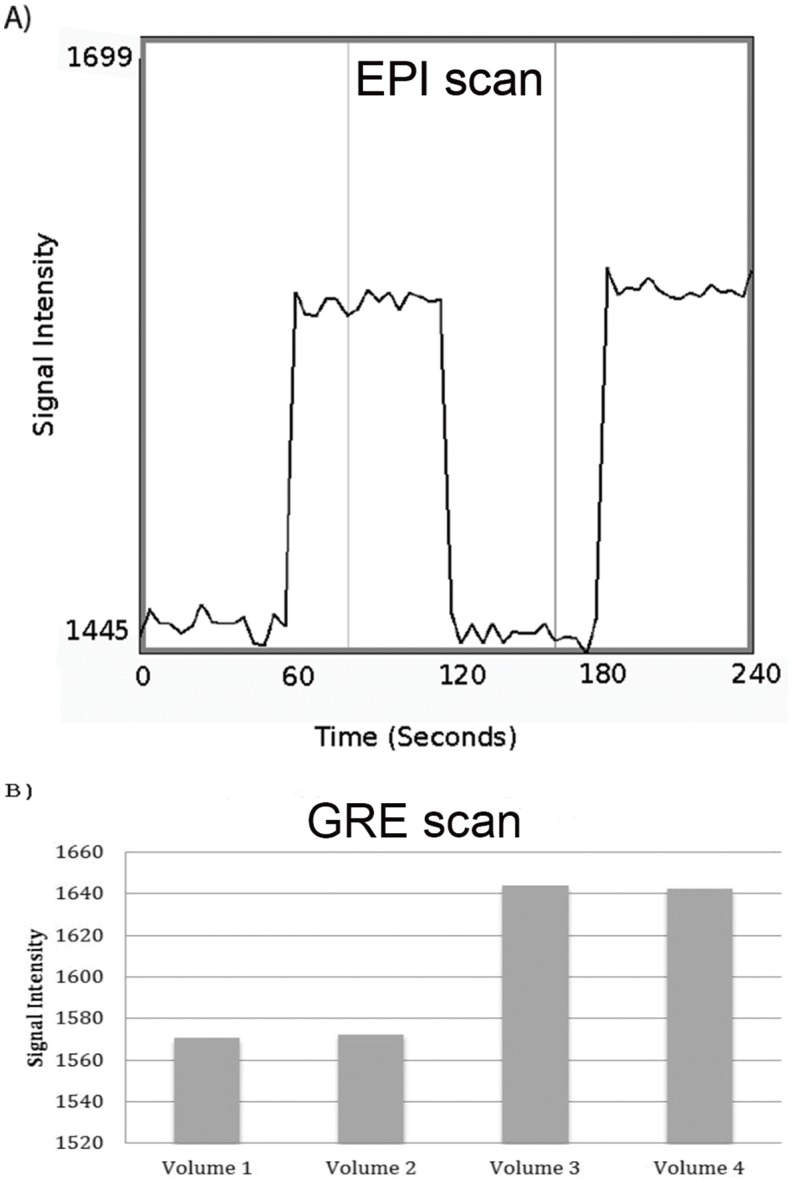
An example of temporal changes in signal for EPI and GRE. Signal changes from a single voxel located in the 1.6% agar wedge, during a simulated fMRI block experiment: A) EPI run with 60 scans, TE = 60 ms, with phantom rotation after every 15 scans. B) GRE run with 4 scans, TE = 30 ms, with one phantom rotation after the 2nd scan.

Fig 5 shows the signal plots of time courses for a single voxel in the 1.6% wedge compartment located in center slice, for the simulated block design fMRI experiment. Fig 5A is taken from an EPI scan, TE = 60ms, after motion correction, with 60 volume acquisitions. There is a clear increase in signal after phantom rotation, from 1450 (arbitrary units) to 1575 and back to 1450, when the phantom is rotated back to its initial position. A voxel in the 1.4% wedge compartments would show a complementary pattern, of a signal drop during rotation. Fig 5B shows the corresponding signal change for a for the GRE scan (TE = 30 ms). Like the EPI, there is a steep increase upon phantom rotation from State A (≈1830 AU) to State B (1915 AU).

### CNR calculations

**Fig 6:**
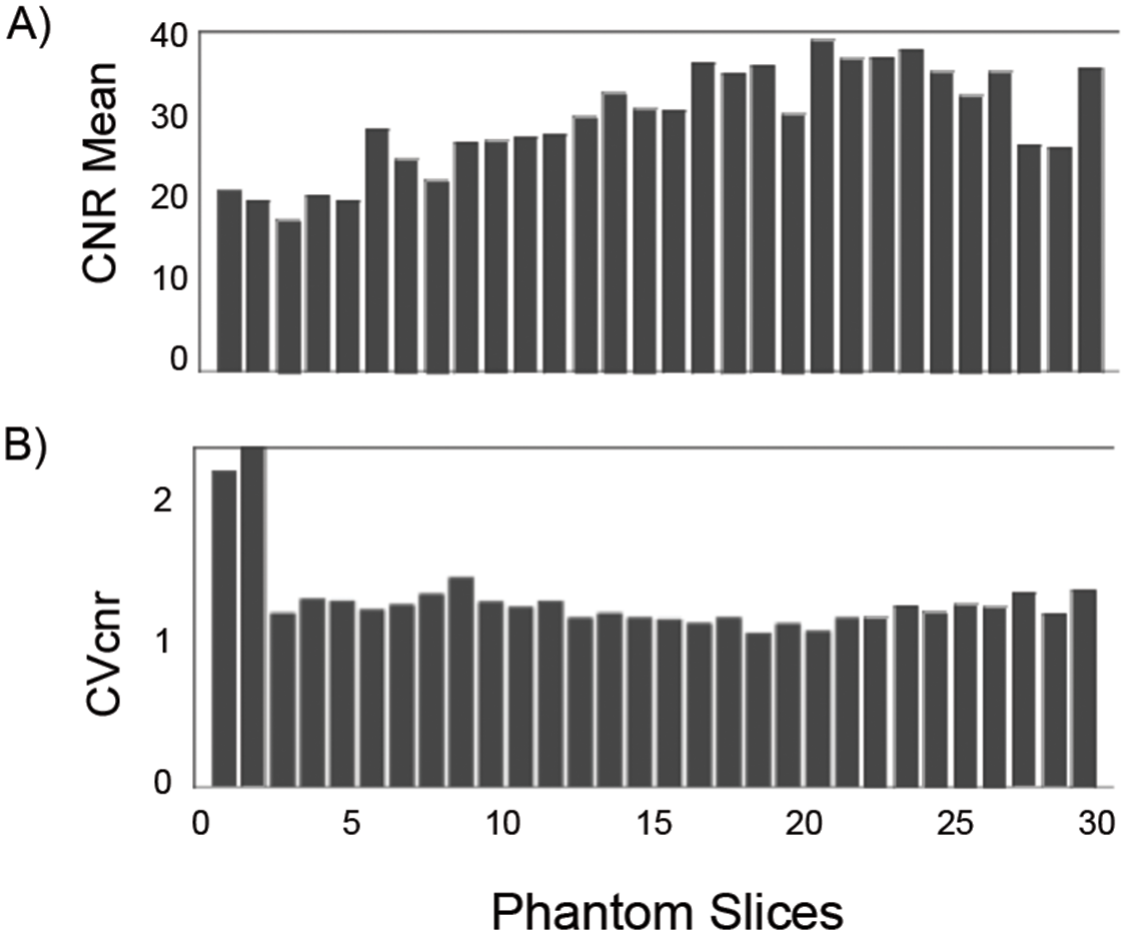
CNR and CV_CNR_ dependence on slice position for GRE scan. A) CNR_mean_ and B) CVcnr plotted across phantom slices for gradient echo scan (TR/TE/FA = 1880 ms/ 30 ms/70°, matrix = 128x128).

**Fig 7:**
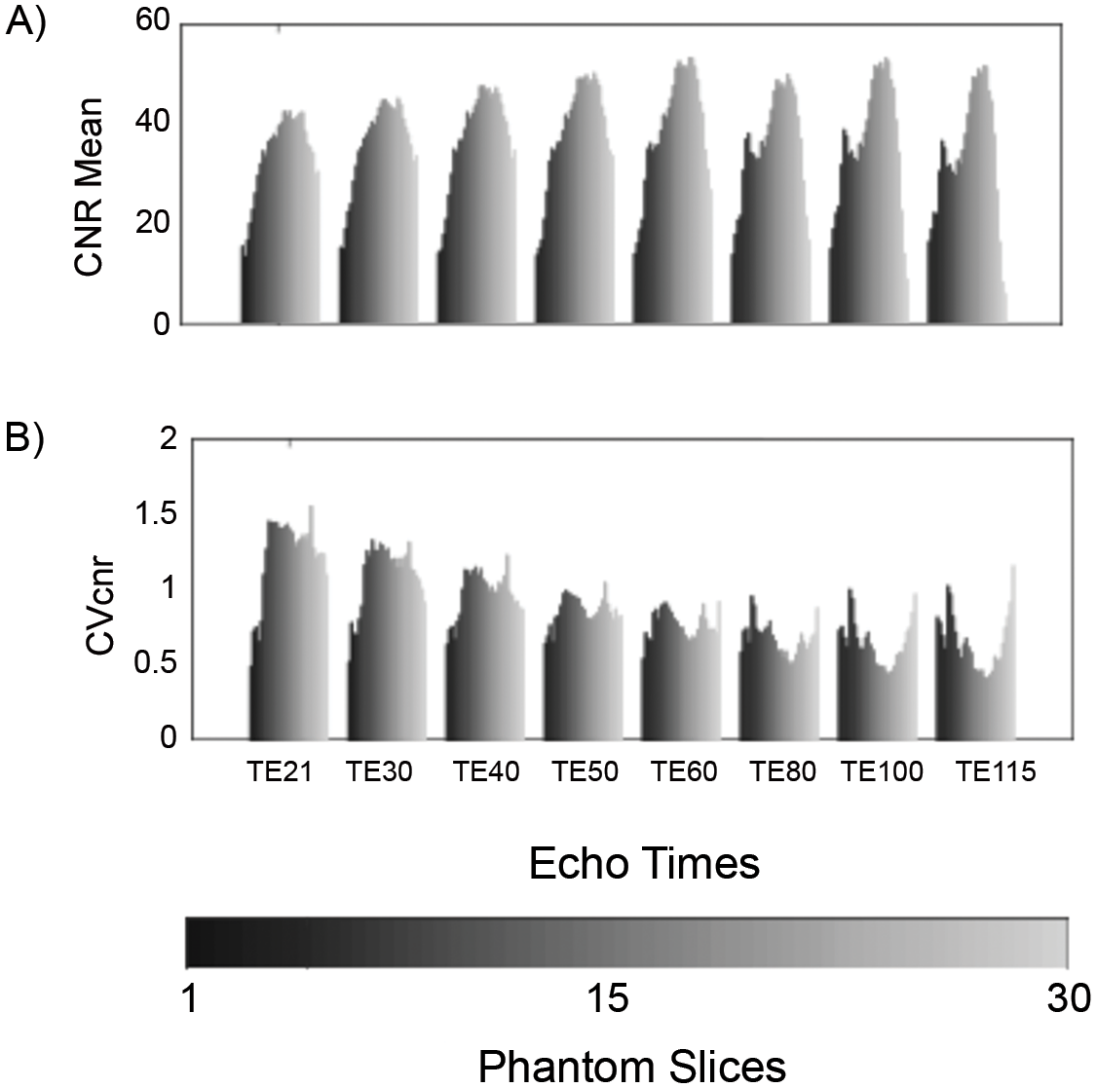
CNR and CV_CNR_ dependence on slice position for EPI scans. A) CNR_mean_ and B) CVcnr plotted across phantom slices for EPI scans at various echo times (TR/TE/FA = 4000ms/21ms-115ms/90°, matrix = 64x64). There is a greater variation of CNR in EPI images compared to the GRE (Fig 6) across the slices and within the slices.

Fig 6A shows the CNR_mean_ measures for the GRE scan which increases from 20 (outer slices) to approximately 40 (at slice 20). Fig 6B shows the CV_CNR_ values which reflects the CNR variation within each of the slices. Except for the first two slices, the CV_CNR_ was found to be close to 1. Assuming the T_2_ values of the gel are relatively uniform (Fig 3), the CNR variation across the slices reflects the added effects of the coil sensitivity, B_1_ effects and B_0_ inhomogeneity. Fig 7 shows the CNR_mean_ and CV_CNR_ for the EPI scans, for different echo times used. The EPI results of Fig 7A are qualitatively similar to GRE scan seen in Fig 6A. However, the variation across the slices is somewhat more pronounced for the EPI scan. Results for each EPI scan at different echo times are grouped together. The grey scale gradient across bars represents the slice positions with dark colored bars indicating the most superior position. The CNR_mean_ (Fig 7A) varies substantially across the slices of the phantom with edge slices nearly half of the peak value. For example, the CNR_mean_ increase from 18 (edge slice) to 50 (slice 20), for an echo time of 50 ms. Additionally, the CNR_mean_ increases with echo time as expected up to approximately 60 ms. The CV_CNR_ values generally vary between 0.5 and 1.5 across the slices and across the echo times studied. The temporal standard deviation σ averaged for all the voxels in each slice was found to be 3.63±0.16, for the TE = 30 EPI scan (~0.2% of the mean signal).

### Phantom stability during storage

The phantom was tested three times using the same scan protocol over 25 days. The gel compartments were found to be intact and the imaging results were found to be qualitatively similar. The mean (± standard deviation) values for the CNR_mean_ for the three EPI (TE = 30 ms) scans over the 25 day (days 1, 12, 25) period were found to be 38.0±5.9, 39.1±4.7, and 39.0±4.6, averaged over 25 slices (slices 6 to 30). The Paired t-tests between all three comparisons (day 1 versus day 12, day 1 versus day 25, and day 12 versus day 25) of CNRmean showed absence of statistical significant differences. The complete table of CNRmean data is provided in the Supporting Information (S1 Table) (p>0.28 for all three comparisons). The percent change in signal between the two states for the Te = 60 ms echo time EPI scan, average over all the slices was found to be 10.6±7.6, 10.8±7.7, and 10.2±7.3, for the three sessions, respectively. In addition, imaging performed at after 1 year, showed a slight increase in T2 of the agar by 2.75%, with no change in standard deviation.

## Discussion

In this study, we have described the use of a rotational phantom to evaluate the MRI system performance and to develop image quality metrics for use in fMRI quality control. The basis of this phantom is the generation of a bimodal signal state for each pixel in the phantom region, for every 15 scans repetitions, over the course of 60 scans, which allows for the evaluation of temporal changes as in a task-based fMRI scan. The phantom is rotated by 45° manually after every 15 acquisitions during the EPI run. The manual rotation, which involves a person turning the phantom from outside the magnet, could potentially introduce motion artifacts due to mechanical imprecision. The phantom was mechanically designed to be balanced, firmly connected to the patient table and be turned precisely with stops to minimize artifacts. We have addressed the rotational imprecision in part using the AFNI based 3dvolreg program to improve registration. In principle, the experiment can also be done by performing the rotation digitally (in post-processing) rather than actually turning the phantom. This would have the disadvantage of mixing in the temporal behavior of two spatially distinct regions into results for one region. Alternately, one could record 60 scans with the phantom rotated once after 30 scans, to minimize the effects of rotation. It is possible that such approaches may be adequate and need to be verified.

Another key challenge in designing an fMRI phantom is the need to generate uniform signal over the usable volume of the phantom. It is desirable to have a uniform phantom composition so that the spatial inhomogeneity of the signal caused by the fMRI pulse sequence is readily detected. In addition, it is also desirable to generate a small but significant difference in T2 weighted signal between the adjacent wedge compartments with material having T1 and T2 close to that of grey matter. The T1 and T_2_ values of the gel used in this phantom were found to be close to that of human grey matter (1470/99 ms) [32]. The slice to slice differences and errors in T2 values as seen in Table 1 of approximately 4% are higher than that found in T2 measurements of homogenous media (typically around 2%), using the same pulse sequence. The higher standard deviations probably result from combining the voxels from four compartments with gels from different batches of preparation, rather than slice to slice differences. Across slices, within each compartment, the values are not expected to vary significantly since each compartment has gel from a single preparation. Also, as seen from Table 1 and Fig 3, an approximate difference of 10% in the T2 values of the two gel compartment provided clear separation of the T2-weighted signal across all the slices in the phantom. The fall-off in signal intensity observed in the outer slices (slices 1–5, 25–30) is consistent with the sensitivity profile of the head-coil used in these experiments and is an added benefit of using a large phantom to assess the effect of coil sensitivity. This is also supported by the finding that the T_1_ and T_2_ values of the compartments are relatively uniform across the slices. In addition, although minor filling defects and air bubbles were noted in the images, the results were found to be reproducible during the 4 week period that the phantom was tested.

The Jaccard index was used to estimate distortion relative to a center-slice MPRAGE image. A potential limitation is that it is not sensitive to shifts in voxels *within* the agar volume. Therefore, the Jaccard index is not the optimal choice to measure spatial distortions within the phantom. However, the Jaccard index could still permit a reproducible estimate of image distortion due the distortion related shifts of the partitions (relative to the MPRAGE image). Although, it appears that an index value of >0.8 offers a qualitatively acceptable image, it unclear what an acceptable limit would be for quality control.

Fig 5, which shows the signal change between the two states (0, 45 degrees rotational positions), reflects the T_2_* signal difference between the 1.4% and 1.6% agar compartments. The percent changes observed (8.5% for EPI and 4.5% for GRE) are consistent with the difference in T_2_ of the two gel compartments (approximately 96 and 106 ms) and TE (60 ms for EPI and 30 ms for GRE). Although a signal change of 6% was found for TE = 30 ms, it is desirable to have an even lower value. The relative percent changes measured using the EPI and GRE sequences reflect the signal deterioration associated with the implementation of EPI pulse sequence and could potentially be used as a figure of merit.

Figs 6 and 7 demonstrate the distribution of CNR over the slices and as a function of TE for EPI and GRE pulse sequences. The CNR_mean_ values shown in Fig 6 for the GRE sequence across slices reflects the combined effects of phantom uniformity, coil performance (B_1_ uniformity and sensitivity), and B_0_ homogeneity. In understanding the increase in CNR_mean_ from 20 (slice 1) to 40 (slice 21) and then a fall-off across the slices, the first two reasons (phantom uniformity and coil performance) are not significant based on the uniform response of the T_2_-weighted signal seen in Fig 3. The rather low variation of the CV_cnr_ across the slices (except for the edge slices) further supports the uniformity of the phantom. Hence the most probable reason for the CNR_mean_ variation across slices in Fig 6 is B_0_ inhomogeneity. The contributions of B_0_ inhomogeneity was not systematically evaluated in this study. Using the automatic shimming routine used in the scanner, the typical water line widths were approximately 50 Hz. In actual QC protocols, a minimal acceptable value for the water line width would need to be agreed upon One of the shortcomings in the estimation of CNR for GRE in this study is the use of only four temporal data points (4 scans taken in the interest of time). A better estimate of standard deviation using more time points would be needed in an actual quality control implementation. Although, a CNR_mean_ of 20 is adequate to detect a 5% signal change in the phantom, it is unclear what the acceptable limit would be for fMRI quality control. The CNR_mean_ variation as a function of TE and slices as shown in Fig 7 provides further information on the performance characteristics of the EPI pulse sequence, over the baseline performance of the system using the GRE sequence. The slice variation of the CNR_mean_ for EPI scans is significantly worse compared to GRE and as expected, worsens with increasing TE, which is consistent with B_0_ inhomogeneity effects. The CNR_mean_ for EPI also depends on the contributions from temporal instabilities, because σ is computed from the input of the 60 EPI acquisitions for each voxel, unlike for the GRE scan. A significant temporal noise would lead to further degradation of CNR_mean_. Also, the minimal slice dependence of σ implies that degradation in CNR_mean_ for the edge slices is not caused by σ differences, but rather from drop in signal intensity. The CNR_mean_ and σ provide a metric for quality control for fMRI, although the acceptable values would have to be generated from multi-site studies. The plateauing of CNR at TE greater than 60 ms is consistent with the expected contrast behavior of material with T_2_* values of 60 and 70 ms. The CNR from two regions with assumed T_2_* values of 95 and 105 ms is expected to peak at 100 ms, assuming the noise is independent of echo time [31,32]. Because physiological noise dominates fMRI scans, it is not clear what level of CNR_mean_ performance in a phantom would be needed for robust fMRI scans. While the phantom and the metrics developed serve as a tool in measuring scanner performance and pulse sequence implementation, many of the metrics are unique to the phantom and are not directly applicable in human scans. They are nonetheless important. For example, measuring an acceptable value of CV_cnr_ in a given MR system with a phantom provides improved confidence in the interpretations of left-right comparisons, such as for left-right fusiform face area studies [33].

One of the drawbacks with this design is the need to turn the phantom during the scan. Adding a remotely controlled rotating mechanism, while possible will add to the cost and complexity. Also, the phantom does not contain lipid regions to depict the effect of inadequate fat suppression. This could be potentially addressed by placing a separate compartment outside the cylinder. Another weakness is that this phantom was not primarily designed for testing spatial homogeneity and distortion and as such will not substitute for a standard quality assurance phantom. Although, the phantom was tested to be stable over few weeks, it is well known that agarose gels are not stable over long periods (months) and hence would need to be modified with different material for longer shelf life. For future studies we propose an alternate strategy of using a doped aqueous solution with MnCl_2_ instead of gel. It is only critical that the T_2_ of the solution be titrated to ~100 ms, with T_1_ being in the 1 second range. Mn^2+^ is primarily a T_2_ reducing ion and has shown to have a R1 and R2 in the range of 8 s^-1^mM^-1^ at 1.5T [34, 35]. Therefore a 0.1 mM aqueous solution of MnCl_2_ could generate a T_1_, T_2_ in the desired range. The experiment would however need to be performed differently to eliminate the potential jiggling artifacts from rotation: 60 EPI acquisitions should be performed without turning the phantom, and then turned slowly by 45°, followed by another 60 EPI acquisitions. The image sets can then be retrospectively sorted to obtain two sets of data for BOLD type of paradigm. This approach using Mn^2+^/Ni^2+^ is expected to provide improved precision in the T1 & T2 distribution in the phantom, and longer shelf-life. An added advantage of using aqueous solutions over gel is also better control of T2 differences and the ability to lower the signal change in the two states from the current value of 6%.

These measurements were made on a routinely maintained 3T Siemens Trio system that is regularly used for fMRI studies. As such, there is reason to believe these metrics are of a scanner in good working order. Despite the lack of accepted thresholds, the phantom also allows comparisons for new pulse sequences. For example, multiband-EPI pulse sequence which allows higher temporal or spatial resolution has been recently reported [36]. However, the extent to which these improvements in spatial or temporal resolution decrease the sequences ability to detect BOLD changes is yet unknown.

In conclusion, we have introduced a novel multi-compartment phantom design to evaluate the MR system performance of T_2_* scan protocols used in fMRI. This agar gel phantom provides a small quantifiable signal change as seen in BOLD fMRI of about 6%, over a head-size imaging volume, combining the advantages of a small temporal phantom with larger static phantoms. The phantom also provides a reproducible signal and T_2_* contrast tested over a 4 week period, and offers a way to measure and compare CNR and distortion across different MRI systems.

## Supporting Information

S1 Table A table of CNR data recorded during a 25 day period. CNR_mean_ values for all the slices in the phantom, for effective echo times from 21 to 120 ms.
(XLSX)
